# Synthetic Site-Selectively Mono-6-*O*-Sulfated Heparan Sulfate Dodecasaccharide Shows Anti-Angiogenic Properties *In Vitro* and Sensitizes Tumors to Cisplatin *In Vivo*

**DOI:** 10.1371/journal.pone.0159739

**Published:** 2016-08-04

**Authors:** Egle Avizienyte, Claire L. Cole, Graham Rushton, Gavin J. Miller, Antonella Bugatti, Marco Presta, John M. Gardiner, Gordon C. Jayson

**Affiliations:** 1 Institute of Cancer Sciences, Faculty of Medical and Human Sciences, The University of Manchester, Manchester M20 4BX, United Kingdom; 2 School of Chemistry and Manchester Interdisciplinary Biocentre, The University of Manchester, Manchester M1 7ND, United Kingdom; 3 Unit of General Pathology and Immunology, Department of Biomedical Sciences and Biotechnology, University of Brescia, Viale Europa 11, 25123 Brescia, Italy; University of Patras, GREECE

## Abstract

Heparan sulphate (HS), a ubiquitously expressed glycosaminoglycan (GAG), regulates multiple cellular functions by mediating interactions between numerous growth factors and their cell surface cognate receptors. However, the structural specificity of HS in these interactions remains largely undefined. Here, we used completely synthetic, structurally defined, alternating *N*-sulfated glucosamine (NS) and 2-*O*-sulfated iduronate (IS) residues to generate dodecasaccharides ([NSIS]_6_) that contained no, one or six glucosamine 6-*O*-sulfates (6S). The aim was to address how 6S contributes to the potential of defined HS dodecasaccharides to inhibit the angiogenic growth factors FGF2 and VEGF_165_, *in vitro* and *in vivo*. We show that the addition of a single 6S at the non-reducing end of [NSIS]_6_, i.e. [NSIS6S]-[NSIS]_5_, significantly augments the inhibition of FGF2-dependent endothelial cell proliferation, migration and sprouting *in vitro* when compared to the non-6S variant. In contrast, the fully 6-*O*-sulfated dodecasaccharide, [NSIS6S]_6_, is not a potent inhibitor of FGF2. Addition of a single 6S did not significantly improve inhibitory properties of [NSIS]_6_ when tested against VEGF_165_-dependent endothelial cell functions.*In vivo*, [NSIS6S]-[NSIS]_5_ blocked FGF2-dependent blood vessel formation without affecting tumor growth. Reduction of non-FGF2-dependent ovarian tumor growth occurred when [NSIS6S]-[NSIS]_5_ was combined with cisplatin. The degree of inhibition by [NSIS6S]-[NSIS]_5_ in combination with cisplatin *in vivo* equated with that induced by bevacizumab and sunitinib when administered with cisplatin. Evaluation of post-treatment vasculature revealed that [NSIS6S]-[NSIS]_5_ treatment had the greatest impact on tumor blood vessel size and lumen formation. Our data for the first time demonstrate that synthetic, structurally defined oligosaccharides have potential to be developed as active anti-angiogenic agents that sensitize tumors to chemotherapeutic agents.

## Introduction

Angiogenesis has been validated as a target for the treatment of multiple solid tumors, including ovarian cancer [1]. The most widely investigated drug, the monoclonal anti-VEGF antibody, bevacizumab, has been evaluated in ovarian cancer where it improved progression free and, in one trial, overall survival [2–5]. Nevertheless, the clinical benefit remains modest, probably because of redundant signaling. Indeed, pre-clinical and clinical studies have shown that progressive disease in VEGF inhibitor-treated cancer can be mediated by FGF2 and SDF-1α [6, 7]; two of the multiple angiogenic cytokines that are critically dependent on heparan sulfate (HS).

The linear glycosaminoglycan heparan sulfate (HS) is covalently linked to specific protein cores at the cell surface or extracellular matrix, forming HS proteoglycans [8]. HS binds and regulates multiple angiogenic growth factors and chemokines (e.g. FGFs, most VEGFs, HGF, HB-EGF, SDF-1α, IL-8) and is an essential regulator of signal transducing protein complexes by acting as a co-receptor (HS/FGFs/FGFRs) or by sequestering the ligands and preventing their interaction with the cognate receptors [8]. HS is produced by nearly all cell types, manifesting variable sulfation patterns that are determined by the level of sulfation at the 2-*O*-position of hexuronate (iduronate or glucuronate) residues and *N*-, 3-*O*- and 6-*O*-positions of *N*-substituted glucosamine, which alternately form HS (9). Highly sulfated disaccharides are found in negatively charged domains [8, 9] that largely engage protein ligands through ionic forces [8, 9].

HS plays a pivotal role in regulating the biological activity of FGFs. Crystallographic and biochemical studies demonstrated the importance of sulfation at specific positions in iduronate and glucosamine which regulates FGF2/FGFR1 complex activity [10–14]. For example, the FGF2-FGFR1-heparin crystal structure showed that heparin forms numerous contacts with FGF2 and FGFR1 and that 6S moieties are of major importance in mediating both interactions [10]. While FGF2 binds to *N*- and 2-*O*-sulfated sequences, 6S residues are necessary for the assembly of tri-molecular signaling complexes that can activate FGFR [10–14]. The number of 6-*O* residues in partially *N*- and 2-*O*-sulfated HS decasaccharides is critical in prolonging activation of FGF2-induced specific signaling pathways [15]. In contrast, other experimental data also suggest that sulfation density and overall charge of highly sulfated HS domains are more important than the precise HS sequence [15–16].

To what extent the structural specificity of HS sequences dictates growth factor activity remains unclear. However, in previous work we showed that the presence or absence of a single 6S at the non-reducing end of an otherwise homogenously 2-*O* and *N*-sulfated dodecasaccharide was an absolute determinant of the oligosaccharide’s potential to inhibit SDF-1α- or IL-8-dependent biological effects [17].

Here, we evaluated the *in vitro* potential of a series of completely synthetic dodecasaccharides [17–19] to inhibit FGF2- and VEGF_165_-mediated angiogenic effects. We show that a single 6-*O*-sulfate at the non-reducing end of an otherwise homogeneously 2-*O*- and *N*-sulfated dodecasaccharide augments the inhibition of FGF2, whereas the homogeneous 6S species, [NSIS6S]_6_, shows little activity against FGF2. Further, we show that [NSIS6S]-[NSIS]_5_ dodecasaccharide is effective when combined with cytotoxic agent cisplatin in reducing tumor xenograft growth through unique effects on tumor vasculature. Thus our work suggests that precise HS-cytokine structure-function relationships are of critical importance in the development of novel anti-angiogenic agents.

## Materials and Methods

### Cell culture

Human Umbilical Vein Endothelial Cells (HUVEC; Lonza, Slough, UK) were cultured in EBM-2 medium supplemented with SingleQuot growth supplements (Lonza) up to passage 7. FGF2-B9 cells were engineered from a parental human endometrial adenocarcinoma cell line HEC-1B to overexpress secreted version of FGF2 as previously described [20]. HEC-1B and FGF2-B9 cells were cultured in RPMI medium (Gibco, Life Technologies, Paisley, UK) supplemented with 10% fetal bovine serum (FBS; Biosera, Uckfield, UK) and non-essential amino acids (Gibco). G418 (500 μg/ml, Invitrogen, Paisley, UK) was added to the medium for culturing of FGF2-B9 cells. HEC-1B and FGF2-B9 cell conditioned media were generated by culturing nearly confluent cell monolayers in EBM-2 media without supplements containing 2% of FBS for forty eight hours. Conditioned media were collected, centrifuged and supernatant stored at -80°C until required.

Ovarian cancer ES2 cell line (ATCC, Manassas, USA) was cultured in RPMI medium supplemented with 10% FBS.

### Endothelial cell *in vitro* assays

HUVEC proliferation, migration, sprouting and tube formation assays were performed as previously described [18, 21]. FGF2 and VEGF_165_ (Lonza) were used at 5–20 ng/ml and 2.5–20 ng/ml concentration, respectively. The treatment with synthetic dodecasaccharides *in vitro* was performed at 1, 10 and 50 μg/ml concentrations.

### Immunoblotting

HUVEC were plated in 6-well plates (1 x 10^5^ cells/well) in EBM-2 medium. Cells were serum-starved in EBM-2 media lacking SingleQuot growth supplements and containing 0.1% FBS for 24 hours. Cells were stimulated with FGF2 (5 ng/ml) or VEGF_165_ (10 ng/ml) for 5 minutes. Preparation of cell lysates and immunoblotting were performed as described [18].

### Binding assays

Ninety six-well MaxiSorp plate (Thermo Fisher Scientific, Scientific Laboratory Supplies, Nottingham, UK) was coated with FGFR1 IIIc-Fc (Arg22-Glu285; R&D, Oxford, UK) or VEGFR2-Fc (R&D) at 4 μg/ml concentration in PBS, or 1% BSA at 4°C overnight. FGF2 (100 ng/ml; R&D) and VEGF_165_ (100 ng/ml; R&D) alone or premixed with dodecasaccharides were added to the plates for 2 hours at room temperature. Following washes with PBS, HRP-conjugated anti-FGF2 or anti-VEGF antibody (R&D) was added for 2 hours at room temperature. After washes, TMB solution was added for 30 minutes. Reactions were stopped with 2M sulphuric acid and optical density was measured at 450 nm.

### FGF2-mediated cell-cell adhesion assay

The assay was performed as previously described [22], with minor modifications. Briefly, wild-type chinese hamster ovary epithelial (CHO-K1) cells were seeded in 24-well plates at 150,000 cells/cm^2^. After 24 hours, cell monolayers were washed with PBS and incubated with 3% glutaraldehyde in PBS for 2 hours at 4°C. Fixation was stopped with 0.1 M glycine, and cells were washed extensively with PBS. Then, HS proteoglycan-deficient FGFR1-over-expressing A745 CHO flg-1A cells (50,000 cells/cm^2^) were added to CHO-K1 monolayers in serum-free medium containing 10 mM EDTA in the absence or presence of FGF2 at 1.66 nM concentration and increasing concentrations of dodecasaccharides. After 2 hours at 37°C, unattached cells were removed by washing twice with PBS and A745 CHO flg-1A cells bound to the CHO-K1 monolayer were counted under an inverted microscope at x125 magnification. Adherent A745 CHO flg-1A cells have rounded morphology and can be easily distinguished from the confluent CHO-K1 monolayer which is detected in a different plane of focus. All experiments were performed twice in triplicate. Data were plotted as a percentage of adherent cells compared to control experiments in the absence of dodecasaccharides.

### Tumor xenografts and treatments

Female Balb/c-NUDE and NSG mice (CR-UK Manchester Institute) were housed in an individually ventilated caging system on a 12-hour light/dark environment maintained at constant temperature and humidity. Mice were fed a standard diet of irradiated feed (Harlan-Teklad, WI, USA) and allowed water *ad libitum*. All procedures were carried out in accordance with UKCCCR guidelines 1999 by approved protocol (Home Office Project license no. 40–3306).

HEC-1B, FGF2-B9 and ES2 cells were grown as subcutaneous xenografts in mice following the injection of 5 x 10^6^ cells into each animal. Each animal group consisted of 6–9 animals. After the formation of a palpable tumor of up to 50 mm^3^ in size HEC-1B tumor-bearing mice were left untreated, while FGF2-B9 tumor-bearing mice were treated with saline s.c., [NSIS6S]-[NSIS]_5_ (160 mg/kg) s.c. b.i.d. or sunitinib p.o. (40 mg/kg) once daily for 21 days. Mice with ES2 xenografts were dosed for 10 days with saline s.c. daily, cisplatin (5 mg/kg) i.p. once a week, [NSIS6S]-[NSIS]_5_ (160 mg/kg) s.c. b.i.d., bevacizumab (15 mg/kg) i.p. twice a week or cisplatin in combination with each of the anti-angiogenic agents.

The volume of tumor xenografts was measured daily. Tumor volume was defined as length x width^2^/2. Tumors were snap-frozen for subsequent analyses.

### Immunofluorescence staining

Tumor sections were fixed with 4% paraformaldehyde for 15 minutes, washed, blocked in 10% goat serum, and incubated with rat anti-mouse CD31 antibody (BD Pharmigen, Oxford, UK) diluted 1:100 in PBS containing 1% BSA for 2 hours at room temperature. Tumor sections were washed and incubated with AlexaFluor568–conjugated donkey anti-rabbit IgG antibody (1:1000; Invitrogen) and Hoechst 33342 (1:1000; Invitrogen) for 1 hour at room temperature. When required, Cy3-conjugated anti-smooth muscle actin α antibody (Sigma, Dorset, UK) diluted 1:200 was used for staining.

To analyse the levels and distribution of phospho-FRS2 in xenografts tumor sections were co-stained for phospho-FRS2 (Y436; R&D) diluted 1:50 and mouse CD31 diluted 1:100 in PBS. After overnight incubation at 4°C, sections were washed and incubated with AlexaFluor488–conjugated donkey anti-mouse IgG antibody (1:800; Invitrogen) and AlexaFluor568–conjugated donkey anti-rabbit IgG antibody (1:800, Invitrogen), respectively. After washing, tumor sections were mounted using ProLong Gold antifade reagent (Invitrogen). Images were acquired with 3D-Histech Mirax scanner (3DHISTECH), viewed with Pannoramic Viewer software (3DHISTECH) and analysed by ImageJ software to determine the number and the size of vessels which was expressed as a number and average size of fluorescent objects per 10,000 pixel of non-necrotic tissue area which was determined by MetaMorph image analysis software (Molecular Devices). Immunofluorescence staining for murine CD31 and SMAα in ES2 xenograft sections was analysed using Definiens software.

### Statistical analysis

Data are expressed as the mean ±SEM. For comparison of groups, the two tailed Student’s *t* test was used. A level of *P* < 0.05 was considered as statistically significant.

## Results

### The effects of dodecasaccharides on FGF2- and VEGF_165_-dependent endothelial cell functions

**Fig 1:**
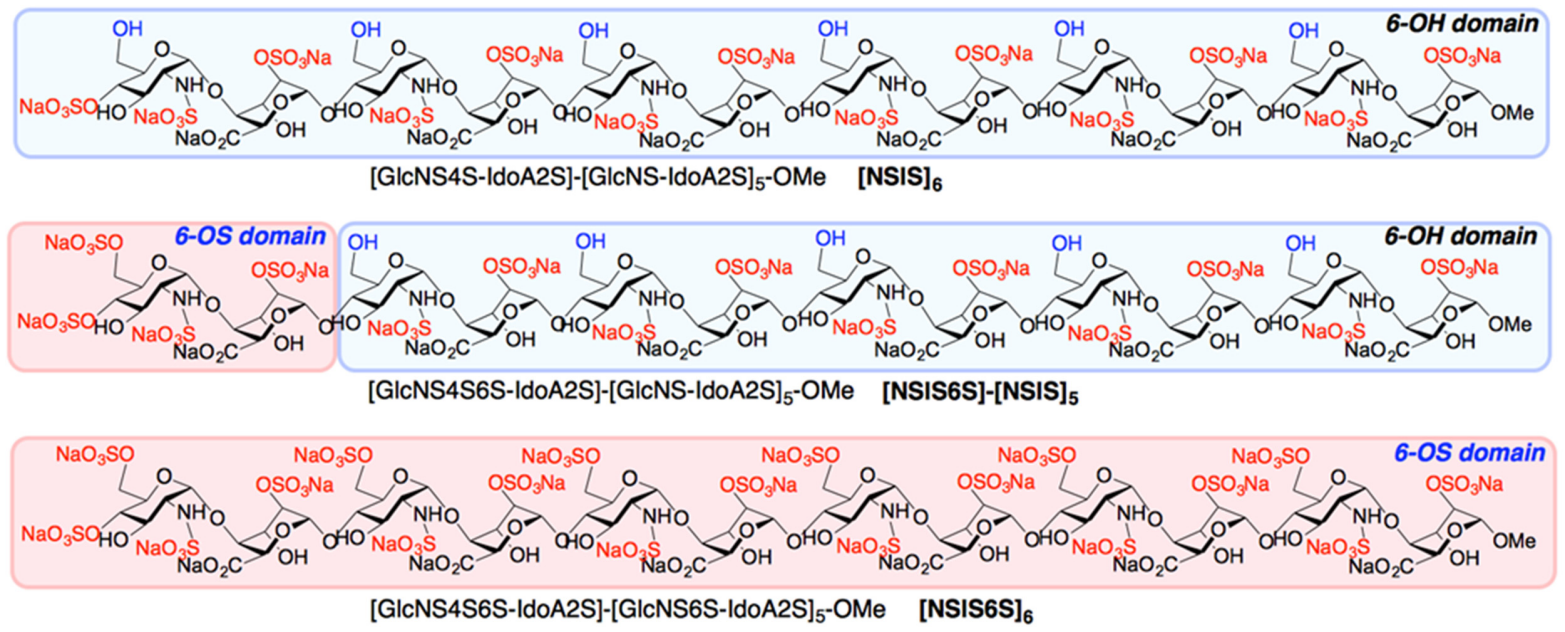
Site-selectively 6-*O*-sulfated dodecasaccharides. Schematic representation of completely non-6-*O*-sulfated ([NSIS]_6_), site-selectively mono-6-*O*-sulfated ([NSIS6S]-[NSIS]_5_) and fully 6-*O*-sulfated ([NS6SIS]_6_) dodecasaccharides. [NSIS]_6_ refers to a dodecasaccharide that consists of 6 alternate *N*-sulfated glucosamine (NS)-Iduronic acid 2-*O*-sulfate (IS) disaccharides. [NSIS6S]-[NSIS]_5_ refers to a dodecasaccharide that contains a 6S moiety in glucosamine of the first disaccharide at the non-reducing end. [NSIS6S]_6_ refers to a dodecasaccharide sulfated at every NSIS disaccharide.

**Fig 2:**
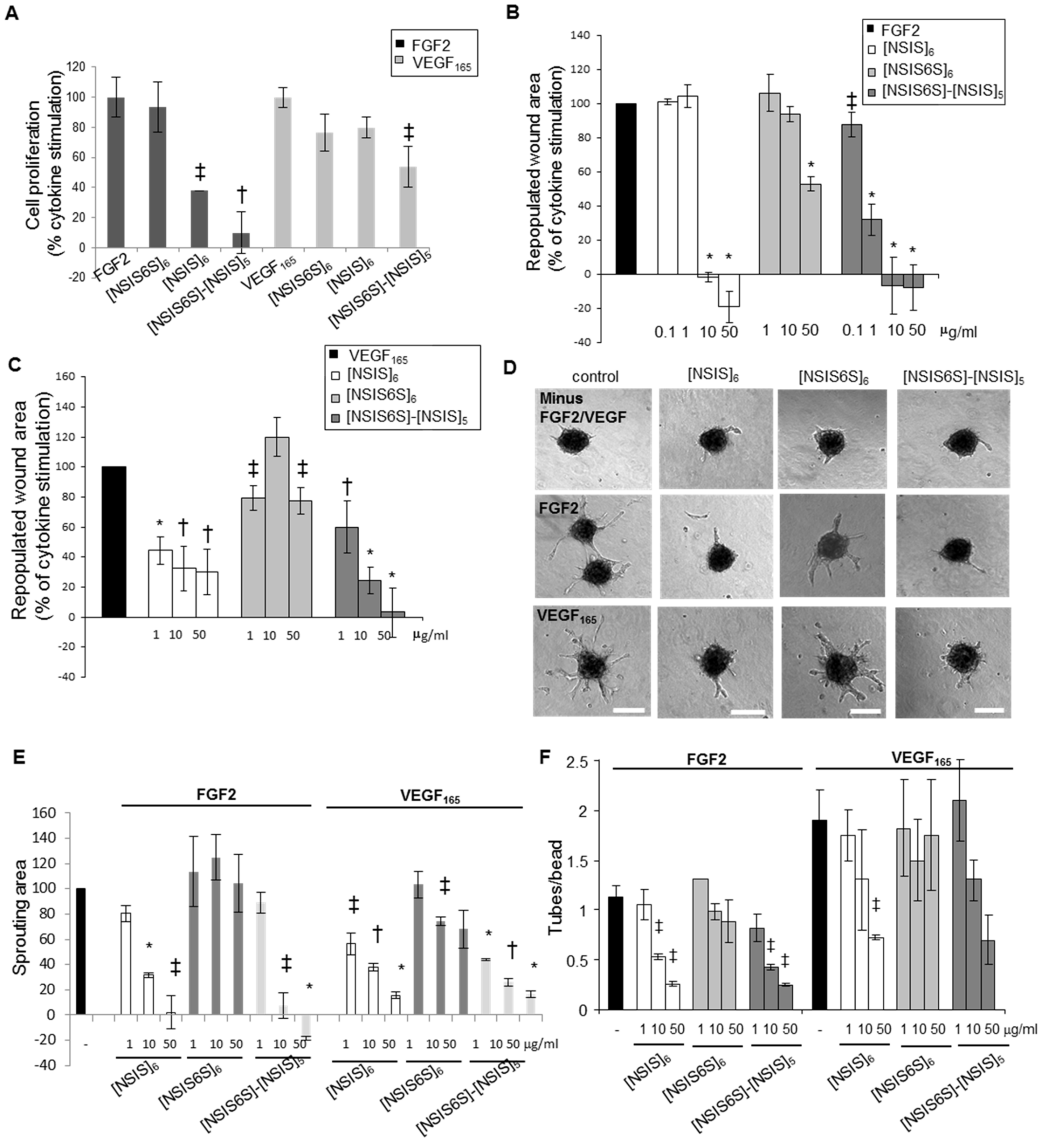
*In vitro* inhibitory potential of site-selectively 6-*O*-sulfated dodecasaccharides. A, dodecasaccharides were tested for effects on FGF2- and VEGF_165_-induced HUVEC proliferation. The treatments were performed for 5 days. Stimulation of proliferation in response to FGF2 (20 ng/ml) and VEGF_165_ (20 ng/ml) is expressed as 100%. Dodecasaccharides were used at 50 μg/ml concentration. The mean ± SEM (n = 3) is shown. †, *P* < 0.01; ‡, *P* < 0.05. B to C, inhibition of HUVEC FGF2- and VEGF_165_-induced migration by dodecasaccharides was tested in wound healing assay. Wounds were created in confluent monolayers of serum-starved HUVEC and FGF2 (B) or VEGF_165_ (C) was added to promote cell migration into the wounds. Dodecasaccharides were used at a range of concentrations starting from 0.1 μg/ml for FGF2 and 1 μg/ml for VEGF_165_ with the highest concentration being 50 μg/ml in all assays. The wound area was measured at the beginning and 24 hours after the treatment. The area repopulated after 24 hours in response to growth factor alone is expressed as 100% (control). The effect of dodecasaccharides is expressed as a percentage of repopulated area induced by a growth factor alone. All experiments were performed three times in triplicates. The mean ± SEM (n = 3) is shown. *, *P* < 0.001; †, *P* < 0.01; ‡, *P* < 0.05. D, HUVEC spheroids were embedded in fibrin gels that were overlaid with either EBM-2 media lacking FGF2 and VEGF_165_, EBM-2 media supplemented with FGF2 (5 ng/ml) or VEGF_165_ (2.5 ng/ml) and EBM-2 media supplemented with FGF2 or VEGF_165_ and dodecasaccharides (50 μg/ml). The treatment was performed for 24 hours. Scale bars represent 200 μm. E, sprouting area in each spheroid was evaluated using Metamorph software where the area of outgrowing sprouts was derived by subtracting the area of a spheroid without sprouts from the total area of a spheroid. The increase of sprouting area after stimulation with FGF2 or VEGF_165_ is expressed as 100% (control). The ability of oligosaccharides to reduce FGF2 and VEGF_165_-induced endothelial cell sprouting is expressed as a percentage of control. 20–30 spheroids were analysed per each experiment and three independent experiments were performed. The values are shown as mean ± SEM (n = 3). *, *P* < 0.001; †, *P* < 0.01; ‡, *P* < 0.05. F, the effect of oligosaccharides on endothelial tube formation was evaluated in three-dimensional fibrin gel bead assay. FGF2 and VEGF_165_ were used at 5 ng/ml concentration. No endothelial tubes developed in the absence of FGF2 and VEGF_165_. Dosing was performed for 5 days. The ratio of average number of endothelial tubes per bead is shown. Three independent experiments were performed each in triplicate. The values are expressed as mean ± SEM (n = 3). *, *P* < 0.001; †, *P* < 0.01; ‡, *P* < 0.05.

Previously we showed that addition of a single 6S moiety at the non-reducing end of an otherwise uniformly 2-*O*- and *N*-sulfated dodecasaccharide converts activity from inhibition of IL-8 to inhibition of SDF-1α [17]. Since FGF2 biological activity crucially depends on 6-*O*-sulfation [11–14], we sought to define the effects of differentially 6-*O*-sulfated dodecasaccharides (Fig 1) on different endothelial cell functions, all of which are crucial in angiogenesis and are regulated by FGF2 and VEGF_165_. FGF2-dependent HUVEC proliferation was inhibited by [NSIS]_6_ and [NSIS6S]-[NSIS]_5_ by 64% and 91%, respectively, demonstrating significantly enhanced inhibitory activity by addition of one 6S. [NSIS6S]-[NSIS]_5_, but not [NSIS]_6_, also inhibited VEGF_165_-dependent cell proliferation by nearly 50% when dosed at 50 μg/ml concentration (Fig 2A). We previously reported that [NSIS6S]_6_ did not inhibit FGF2- or VEGF_165_-mediated cell proliferation (Fig 2A; 19) and the current evaluation alongside [NSIS]_6_ and [NSIS6S]-[NSIS]_5_ confirms the effect of uniform 6-*O*-sulfation in abrogating inhibitory effects.

When examined for effects on FGF2-induced HUVEC migration into the wounds *in vitro*, [NSIS6S]-[NSIS]_5_ was more potent than [NSIS]_6_ in inhibiting FGF2-induced HUVEC migration at 1 μg/ml concentration (Fig 2B). Similarly, VEGF_165_-induced cell migration was reduced by [NSIS6S]-[NSIS]_5_ more significantly than by [NSIS]_6_, although at a much higher concentration (50 μg/ml; Fig 2C). [NSIS6S]_6_ was largely ineffective (Fig 2B and 2C), confirming our prior evaluations (19).

Endothelial cell sprouting is an important endothelial tip cell function during sprouting angiogenesis. In HUVEC sprouting assay, [NSIS6S]-[NSIS]_5_ inhibited FGF2-induced sprouting in fibrin gels by 92%, while [NSIS]_6_ inhibited sprout formation by 68% at 10 μg/ml (Fig 2D and 2E), thus demonstrating a better anti-angiogenic efficacy of [NSIS6S]-[NSIS]_5_. Fully 6S sulfated dodecasaccharide showed no effect on FGF2-induced sprouting (Fig 2E). Both [NSIS6S]-[NSIS]_5_ and [NSIS]_6_ inhibited VEGF_165_-induced sprouting with similar potency, while [NSIS6S]_6_ showed poor inhibitory potential (Fig 2E). When tested in assays of FGF2- and VEGF_165_-dependent tube formation in three-dimensional bead assay in fibrin gels, [NSIS6S]-[NSIS]_5_ inhibited tube formation by 25% and [NSIS]_6_ had no effect at 1 μg/ml concentration (Fig 2F). Differences between inhibitory potential of [NSIS6S]-[NSIS]_5_ and [NSIS]_6_ were less evident in VEGF_165_-induced tube formation (Fig 2F) and [NSIS6S]_6_ showed no effect in this assay (Fig 2F).

Together these data clearly demonstrate that an additional single 6-*O*-sulfate group in glucosamine at the non-reducing end of [NSIS]_6_ increases the potency of dodecasaccharides in targeting all FGF2 regulated biological effects in endothelial cells, while minimally improving effects against VEGF_165_ regulated functions.

### Dodecasaccharides display structure-dependent specificity in inhibiting FGF2/FGFR1 complex formation

**Fig 3:**
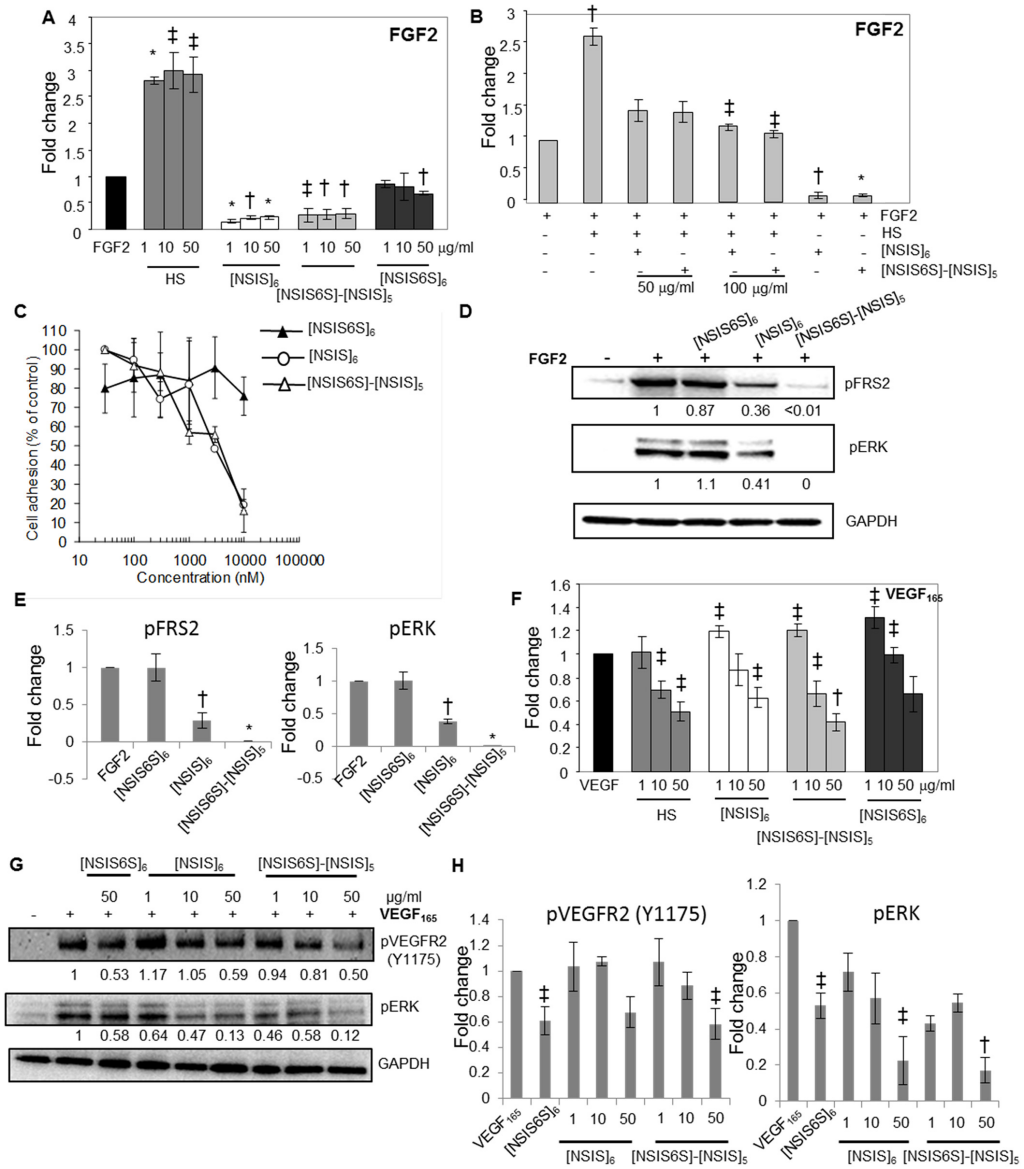
Impact of dodecasaccharides on FGF2/FGFR1 and VEGF/VEGFR2 complex formation. A, levels of FGF2 bound to FGFR1 IIIc-Fc–coated plates were measured by ELISA. Binding of FGF2 to FGFR1 is expressed as 1 (control). Fold change in FGF2 binding to FGFR1-coated plates in the presence of HS or specific dodecasaccharides at increasing concentrations as compared to control is shown. Each experiment was performed twice in triplicate. The data are presented as the mean ± SD (n = 2). *, *P* < 0.001; †, *P* < 0.01; ‡, *P* < 0.05. B, the ability of [NSIS]_6_ and [NSIS6S]-[NSIS]_5_ to compete with HS (1 μg/ml) for binding of FGF2 to FGFR1. FGF2 was premixed with HS alone (1 μg/ml), HS and [NSIS]_6_ or [NSIS6S]-[NSIS]_5_ (50 and 100 μg/ml) or dodecasaccharides alone (50 μg/ml). FGFR1-bound FGF2 was detected by ELISA. FGF2 binding to FGFR1 in the absence of HS and dodecasaccharides is expressed as 1 (control). The data were derived from two independent experiments performed in triplicate and shown as the mean ± SD. *, *P* < 0.001; †, *P* < 0.01; ‡, *P* < 0.05. C, A745 CHO flg-1A cells were added to the wild type CHO-K1 monolayers in serum-free medium containing FGF2 in the absence or presence of indicated dodecasaccharides. Cells adherent to the monolayer were counted following incubation for 2 hours. The data are presented as a percentage of cell binding in the absence of dodecasaccharides (100%). Each point is the mean ± SD of three independent experiments performed in triplicate. D, serum-starved HUVEC were stimulated with FGF2 (20 ng/ml) for 10 min in the absence or presence of indicated dodecasaccharides. Phosphorylated FRS2 and ERK were detected by Western blotting. Total protein loading levels were visualized by probing with the anti-GAPDH antibody. Stimulation with FGF2 alone is expressed as 1. Normalized fold change in the intensities of bands as compared to FGF2 stimulation alone is shown below each blot as determined by densitometric analysis. E, densitometric evaluation of the intensities of bands combined from an independent experiment performed as in D and an experiment shown in D. Fold change in phosphorylated FRS2 and ERK levels in response to FGF2 stimulation in the absence and presence of oligosaccharides is shown. Values represent the mean ± SD (n = 2). *, *P* <0.0001 †, *P* < 0.01. F, levels of VEGF_165_ bound to VEGFR2-Fc were measured using VEGF-specific ELISA. VEGF_165_ binding to VEGFR2-coated plate in the absence of HS or dodecasaccharides is expressed as 1. Two independent experiments were performed in triplicate and the data are presented as the mean ± SD. †, *P* < 0.01; ‡, *P* < 0.0001. G, inhibition of VEGF_165_-induced phosphorylation of VEGFR2 and ERK. Serum-starved HUVEC were stimulated with VEGF_165_ (20 ng/ml) for 5 min in the absence or presence of dodecasaccharides at indicated concentrations. Phospho-VEGFR2 (Y1214 and Y1175) and phospho-ERK were detected by immunoblotting with the respective antibodies. GAPDH levels show equal total protein levels. The intensities of bands for phospho-VEGFR2, phospho-ERK and GAPDH were analysed by densitometry. Phospho-VEGFR2 and phospho-ERK levels were normalized to GAPDH levels. Stimulation with VEGF_165_ alone is expressed as 1. Fold change in the intensities of phosphorylated VEGFR2 and ERK upon each treatment is shown below the blots. H, average values of band intensities generated by densitometric analysis of bands shown in G and those from another independent experiment are shown. Numbers on the horizontal axis show different concentrations of each oligosaccharide. The mean ± SD (n = 2) is shown. †, *P* <0.01 ‡, *P* < 0.05.

Previous studies and our current data suggest that [NSIS]_6_ and [NSIS6S]-[NSIS]_5_ impact on FGF2-dependent endothelial cell functions through competitive inhibition. To investigate this further we tested whether dodecasaccharides modulate FGF2 and FGFR1 complex formation. A significant increase in FGF2 binding to FGFR1-Fc-coated plates was observed when FGF2 was preincubated with native HS (Fig 3A). [NSIS6S]-[NSIS]_5_ and [NSIS]_6_ inhibited FGF2 binding to FGFR1-Fc with similar efficiency, while [NSIS6S]_6_ had a minimal effect at 50 μg/ml concentration (Fig 3A).

To determine if dodecasaccharides prevent HS-mediated tri-molecular complex formation we admixed FGF2 with HS at 1 μg/ml and [NSIS6S]-[NSIS]_5_ and [NSIS]_6_ at 50 and 100 μg/ml concentrations. As shown in Fig 3B, dodecasaccharides reduced HS-mediated FGF2 binding to FGFR1-Fc by nearly 50%, whereas in the absence of HS FGF2 binding to its receptor was inhibited by approximately 90% showing that dodecasaccharides compete with HS for binding to FGF2 and prevent its binding to the receptor We confirmed these results in a previously validated and published cell-cell adhesion assay designed to test FGF2 binding to FGFR1 [22]. HSPG- and FGFR-deficient FGFR1-transfected A745-CHO *flg*-1A cells were allowed to adhere to a monolayer of HSPG-containing CHO-K1 cells in the presence of FGF2 alone or FGF2 admixed with the dodecasaccharides. As expected, [NSIS6S]-[NSIS]_5_ and [NSIS]_6_, but not [NSIS6S]_6_, inhibited FGF2-mediated cell-cell interaction by approximately 80% (Fig 3C).

Next, we investigated the impact of defined dodecasaccharides on FGFR signaling by quantifying the levels of phosphorylated FRS2 and ERK, the downstream effectors of FGFR signaling. [NSIS6S]-[NSIS]_5_ was the most potent inhibitor of FGF2-induced FRS2 and ERK phosphorylation, while [NS6SIS]_6_ did not affect phospho-FRS2 and phospho-ERK levels (Fig 3D and 3E). Despite similar efficiency of tri-molecular complex formation involving FGF2, FGFR1 and [NSIS6S]-[NSIS]_5_ or [NSIS]_6_, FGFR signaling was most affected by [NSIS6S]-[NSIS]_5_, suggesting that one 6S does not impair the binding efficiency but has a profound effect on signaling.

All dodecasaccharides, independently of structure, inhibited the binding of VEGF to VEGFR2 at the highest concentration but the degree of inhibition was lower than for FGF2 (Fig 3F). Similarly, [NSIS6S]-[NSIS]_5_ and [NSIS]_6_ were equally effective inhibitors of VEGF_165_-induced VEGFR2 and ERK phosphorylation (Fig 3G and 3H). These data show that the single non-reducing end 6S has an impact on FGF2/FGFR signaling but has little effect on VEGF/VEGFR2 signaling in endothelial cells.

### The effect of 6-*O*-sulfation in inhibiting cancer cell secreted FGF2

**Fig 4:**
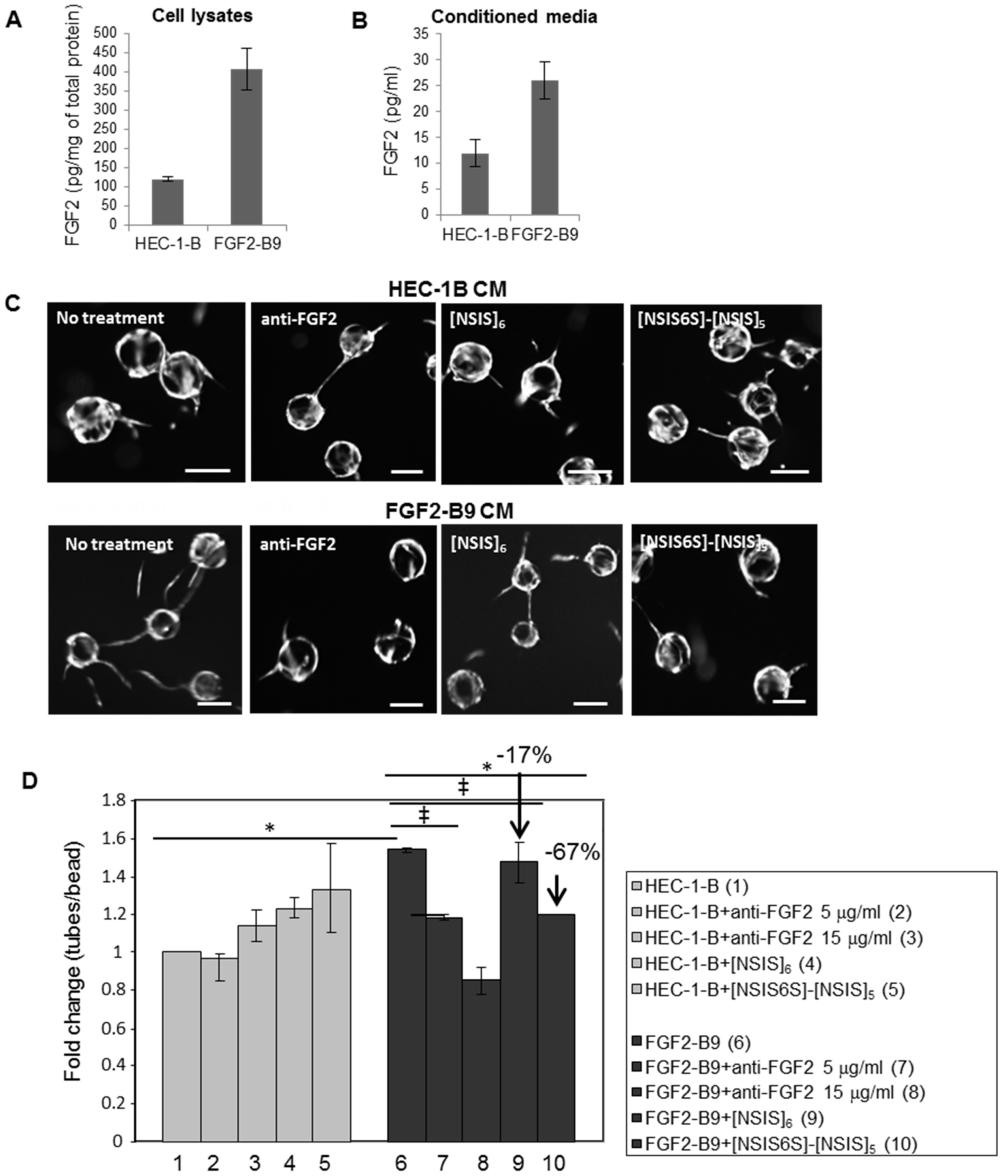
[NSIS6S]-[NSIS]_5_ inhibits FGF2 secreted from cancer cells. A to B, FGF2 concentration in HEC-1-B and FGF2-B9 cell lysates and conditioned medium was determined by ELISA. Two independent experiments were performed and the data are expressed as the mean ± SD. C, angiogenic potential of conditioned media collected from HEC-1-B and exogenous FGF2 overexpressing HEC-1-B cell line FGF2-B9 was evaluated in three dimensional fibrin gel HUVEC bead assay. Conditioned medium collected from HEC-1-B cells induced minimal endothelial tube outgrowth insensitive to blocking anti-FGF2 antibody or treatment with anti-angiogenic dodecasaccharides [NSIS]_6_ and [NSIS6S]-[NSIS]_5_ (upper panel). Treatment with FGF2-B9 conditioned medium resulted in increased outgrowth of endothelial tubes which was reduced by treatment with anti-FGF2 antibody, [NSIS]_6_ and [NSIS6S]-[NSIS]_5_. Scale bars represent 100 μm. D, quantification of a number of endothelial tubes per bead as shown in C. The number of tubes per bead induced by HEC-1-B conditioned medium is expressed as 1 (control). The effect of FGF2-B9 conditioned medium and all treatments is shown as fold change compared to the control. Two independent experiments were performed in triplicate. The data are expressed as the mean ± SD (n = 2). *, *P* < 0.001; ‡, *P* < 0.05.

Since [NSIS6S]-[NSIS]_5_ was the most potent inhibitor of FGF2-dependent HUVEC proliferation, migration and sprouting (Fig 2), we investigated the degree to which [NSIS6S]-[NSIS]_5_ reduces endothelial tube formation induced by conditioned medium generated by endometrial cancer cells expressing exogenous secreted form of FGF2 (FGF2-B9). The concentration of FGF2 was 4- and 2.5-fold higher in FGF2-B9 lysates and conditioned medium, respectively, when compared to the parental HEC-1-B cell line (Fig 4A and 4B). FGF2-B9 conditioned medium increased HUVEC tube formation in fibrin gels 1.5-fold when compared to the medium collected from HEC-1-B cells (Fig 4C and 4D). Next, we investigated whether the increase in tube formation was FGF2 dependent. FGF2 neutralizing antibody reduced tube formation in a dose-dependent manner, where the average number of tubes per bead was reduced to that seen in HEC-1-B conditioned medium at 15 μg/ml concentration (Fig 4C and 4D). [NSIS6S]-[NSIS]_5_ reduced the tube per bead ratio induced by FGF2-B9 conditioned medium by 67% (Fig 4D; the value of the bar 10 compared to that of the bar 6 after substraction of the value of control bar 1), whereas [NSIS]_6_ only reduced this by 17% (Fig 4D; the value of the bar 9 compared to the bar 6 after substraction of control value). Dodecasaccharides did not affect HUVEC tube formation in HEC-1-B conditioned medium (Fig 4C and 4D).

In summary, our *in vitro* data demonstrate that [NSIS6S]-[NSIS]_5_ is a more potent inhibitor of FGF2-dependent endothelial cell functions and signaling than [NSIS]_6_ and that full 6-*O*-sulfation on a 2-*O*- and *N*-sulfated backbone results in a complete loss of inhibitory activity against FGF2.

### [NSIS6S]-[NSIS]_5_ inhibits FGF2-dependent blood vessel formation in tumors

Angiogenesis *in vitro* assays that are designed to test numerous endothelial cell functions involved in the formation of new blood vessels demonstrated that [NSIS6S]-[NSIS]_5_ was more potent than [NSIS]_6_ in inhibiting FGF2-dependent endothelial cell functions (Figs 2–4). Therefore, we selected [NSIS6S]-[NSIS]_5_ for investigation of its anti-angiogenic properties *in vivo*. Considering pharmacokinetic profile of synthetic dodecasaccharides [23], large amounts of dodecasaccharides are required for efficacy experiments *in vivo*, which presents a considerable challenge. Therefore, we performed large-scale synthesis of only one dodecasaccharide, [NSIS6S]-[NSIS]_5_, that was the most potent in inhibiting endothelial cell functions.

**Fig 5:**
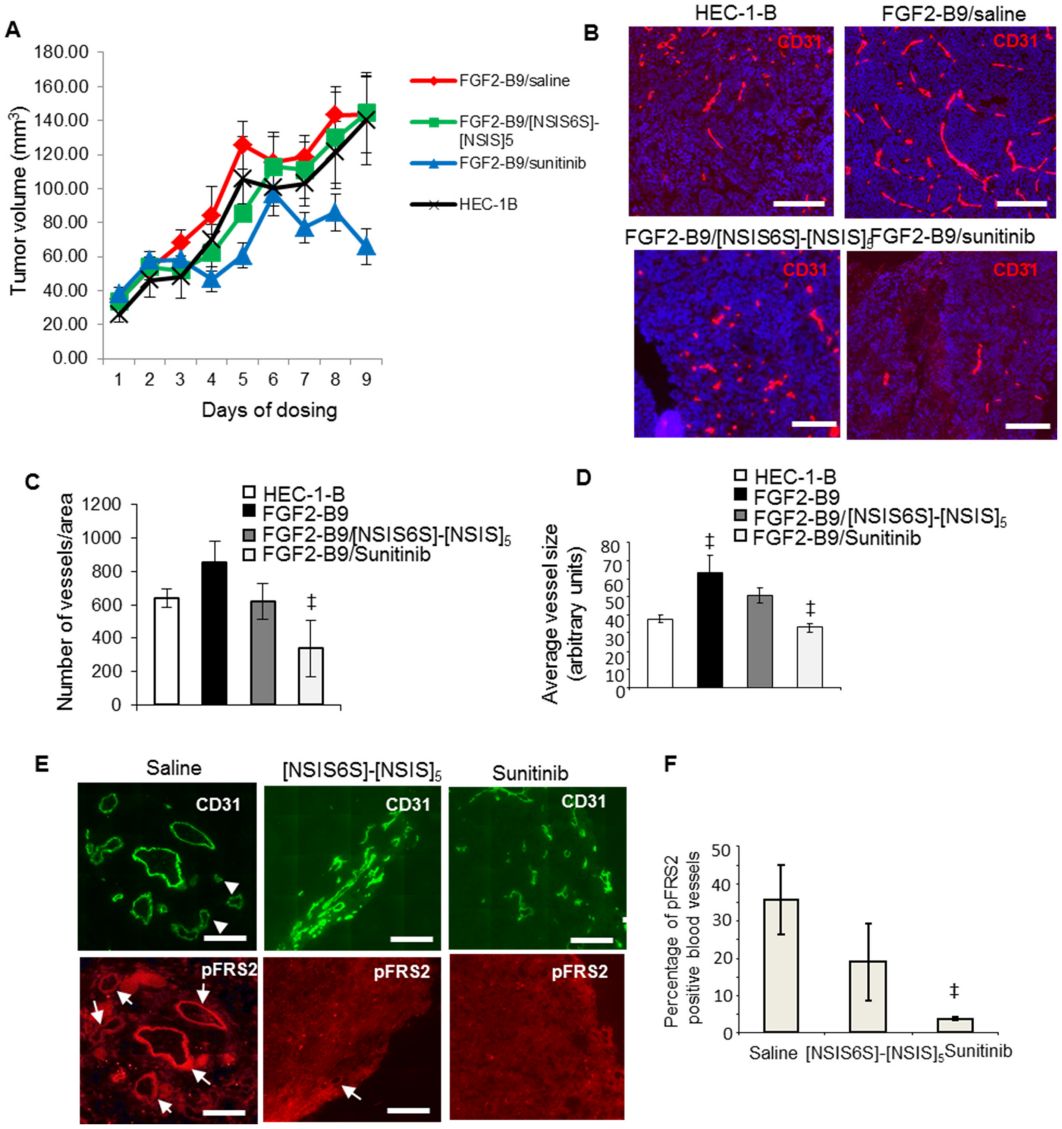
[NSIS6S]-[NSIS]_5_ inhibits FGF2-induced tumor blood vessel formation. A, tumor xenografts were established subcutaneously from HEC-1-B and FGF2-B9 cell lines in female Balb/c-NUDE mice (n = 9) and allowed to grow to a volume of approximately 50 mm^3^ before starting the dosing. HEC-1-B tumor-bearing mice were left untreated, while FGF2-B9 tumor-bearing mice were treated with saline (daily), [NSIS6S]-[NSIS]_5_ (160 mg/kg b.i.d) or sunitinib (40 mg/kg daily) for 9 days. Tumor volume was monitored every day for 9 days. B, two hours after the last dose, animals were sacrificed and tumor sections were processed for immunofluorescence staining with anti-mouse CD31 antibody to visualize tumor vasculature (red). Nuclei were visualized with Hoechst staining (blue). Scale bars represent 250 μm. C, number of vessels in each tumor section was evaluated using ImageJ software where each vessel represents a fluorescent object visualized by anti-CD31 staining. The number of vessels was normalized per area of non-necrotic tumor tissue. Five tumor sections from each of the nine tumors from each treatment group were analyzed. The data are presented as the mean ± SEM (n = 9). D, average vessel size was derived from the analysis of a size of CD31-stained blood vessels in each tumor section using Image J programme. Sections from each treatment group consisting of 9 tumors were analyzed. The data are shown as the mean ± SEM (n = 9). ‡, *P* < 0.05. E, phospho-FRS2 in blood vessels was visualized through immunofluorescence staining of tumor xenograft sections with antibodies against phospho-FRS2 and CD31. White arrows indicate blood vessels that are positive for phospho-FRS2 staining. Arrowheads show blood vessels that are negative for phospho-FRS2 staining. Scale bars represent 200 μm. F, phospho-FRS2 positive blood vessels were counted in a tumor section derived from each tumor. Nine tumors were examined in each treatment group. Percentage of phospho-FRS2 positive blood vessels relative to total CD31-positive vessels is expressed as the mean ± SEM (n = 9). ‡, *P* < 0.05.

We established tumor xenografts from HEC-1-B and FGF2 overexpressing FGF2-B9 cell lines and treated FGF2-B9 xenograft-bearing mice with saline, [NSIS6S]-[NSIS]_5_ and sunitinib, an inhibitor of VEGFR2, PDGFR and FGFRs. Although tumor growth was unaffected in animals treated with [NSIS6S]-[NSIS]_5_ (Fig 5A), microvessel density following evaluation of staining for murine endothelial cell marker CD31 was reduced to the levels seen in HEC-1-B tumor xenografts (Fig 5B and 5C), suggesting that FGF2-dependent angiogenesis in FGF2-B9 xenografts was effectively targeted by [NSIS6S]-[NSIS]_5_. FGF2-B9 tumors developed larger blood vessels than HEC-1-B tumors as a consequence of FGF2 overexpression and [NSIS6S]-[NSIS]_5_ reduced the average vessel size by 51% in FGF2-B9 xenografts (Fig 5D).The fact that sunitinib reduced tumor growth (Fig 5A) as well as FGF2-dependent and -independent vasculature formation in FGF2-B9 tumors (Fig 5B and 5C) suggests that dodecasaccharide inhibitory potency against VEGF is insufficient to slow tumour growth through inhibition of angiogenesis or off-target effects of sunitinib are contributing to reduced tumour growth.

We co-stained tumor sections for phospho-FRS2 and murine CD31 to determine if FGF2 signaling was affected by [NSIS6S]-[NSIS]_5_ treatment in specific cell types in tumors. The dodecasaccharide reduced the number of phospho-FRS2-positive blood vessels by 47% (Fig 5E and 5F), showing that [NSIS6S]-[NSIS]_5_ is an effective inhibitor of FGF2-mediated tumor angiogenesis.

### Synergistic inhibitory effect of [NSIS6S]-[NSIS]_5_ in combination with cisplatin in ovarian cancer

**Fig 6:**
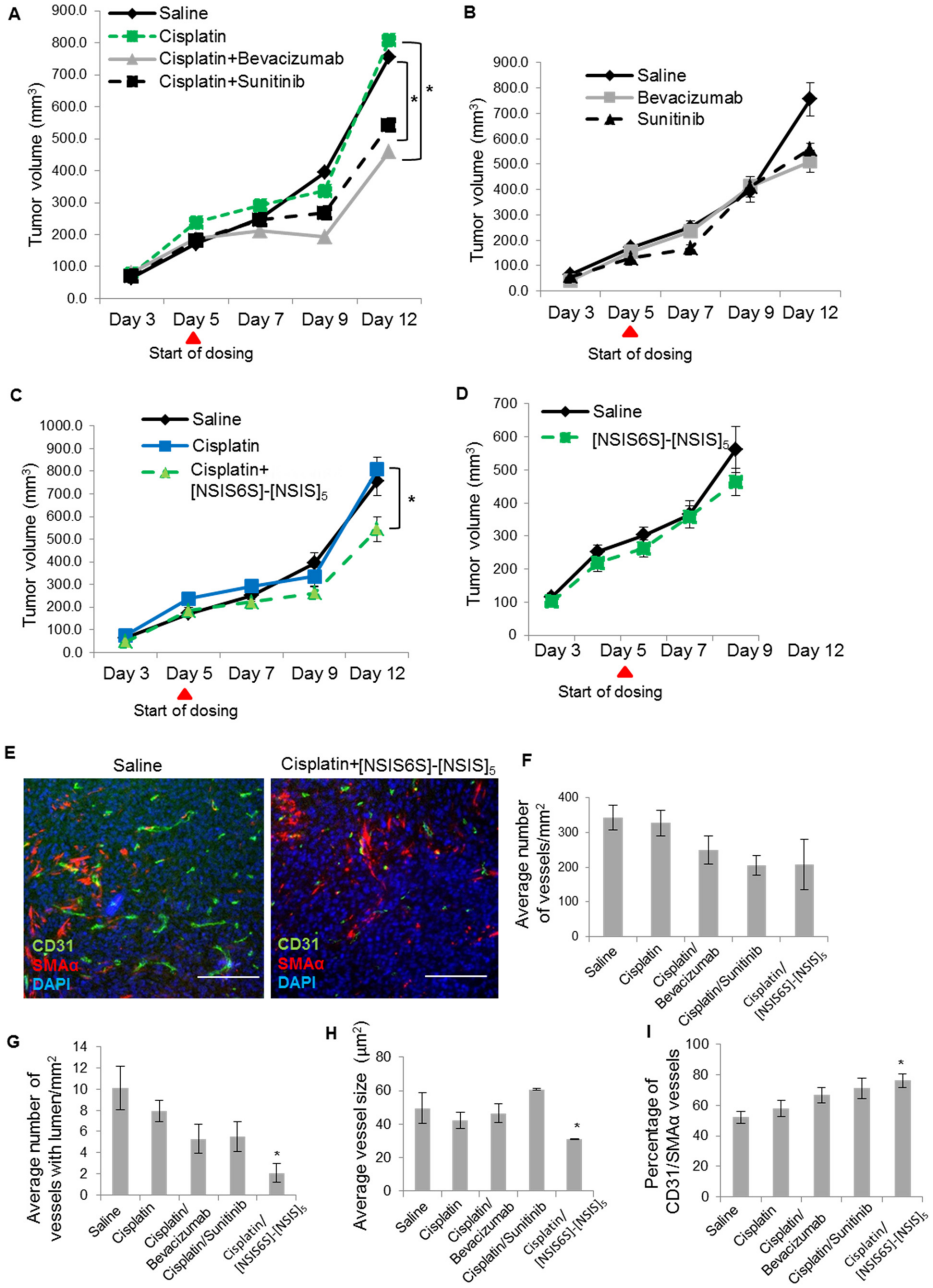
Anti-angiogenic activity of [NSIS6S]-[NSIS]_5_ in combination with cisplatin in ovarian cancer model. A to D, six tumor xenografts were established from ES2 ovarian cancer cell line in each treatment group. ES2 tumor-bearing mice were treated for 7 days with saline (daily), cisplatin (10 mg/kg, once a week) and a combination of cisplatin and bevacizumab (15 mg/kg, twice a week) or sunitinib (40 mg/kg, daily) (A); saline, bevacizumab and sunitinib as single agents at doses indicated in A (B); saline, cisplatin (10 mg/kg, once a week) and a combination of cisplatin and [NSIS6S]-[NSIS]_5_ (160 mg/kg, b.i.d.) (C); saline and [NSIS6S]-[NSIS]_5_ as a single agent at 160 mg/kg b.i.d. (D). *, *P* < 0.05. E, Tumor sections were stained with antibodies against murine CD31 and SMAα to visualise vasculature and perivascular mural cells, respectively. Scale bars, 200 μm. F to I, microvascular density (F), number of vessels with lumen (G), average vessel size (H) and vessel maturity (I) were determined using Definiens software which analyzed images of tumor sections immunostained for CD31 and SMAα. Vessel maturity was determined as a percentage of CD31- and SMAα-positive blood vessels. The data are expressed as the mean ± SEM (n = 6). *, *P* < 0.05.

Anti-angiogenic agents are often co-administered with cytotoxic therapy in the clinic [2–5]. Since we demonstrated that [NSIS6S]-[NSIS]_5_ inhibited FGF2-induced endothelial cell functions, *in vitro*, and targeted FGF2-dependent angiogenesis *in vivo*, we tested its activity *in vivo* in combination with cisplatin. In ES2 ovarian cancer xenograft tumors, cisplatin did not affect tumor growth, whereas when combined with bevacizumab and sunitinib tumor volume was reduced by 44% and 33%, respectively, over 7 days (Fig 6A). Bevacizumab and sunitinib were slightly less effective when dosed as single agents (Fig 6B). The cisplatin/[NSIS6S]-[NSIS]_5_ treatment decreased tumor volume by 33% (Fig 6C) which was as effective as combinatorial treatments with bevacizumab or sunitinib and cisplatin (Fig 6A). [NSIS6S]-[NSIS]_5_ alone was less effective (Fig 6D). Treatments were terminated after 10 days of dosing, since ES2 xenografts are fast growing and aggressive tumours which reach a maximum allowed volume within 10–14 days.

Treated tumors were examined to enumerate microvessel density (Fig 6E). Tumors from animals dosed with bevacizumab, sunitinib and [NSIS6S]-[NSIS]_5_ in combination with cisplatin showed a similar degree of reduction in microvessel density (Fig 6F), but only cisplatin/[NSIS6S]-[NSIS]_5_ treatment resulted in a statistically significant reduction of blood vessels with concomitant reductions in the lumen and the average vessel size (Fig 6G and 6H). Treatment with cisplatin and [NSIS6S]-[NSIS]_5_ resulted in the greatest number of mature vessels as determined by co-staining of CD31 and mural cell marker SMAα (Fig 6I).

Taken together the data show that [NSIS6S]-[NSIS]_5_ sensitizes ES2 tumors to cisplatin treatment to the same extent as clinically approved anti-angiogenic agents bevacizumab and sunitinib. The mechanism through which [NSIS6S]-[NSIS]_5_ impacts on tumor angiogenesis is different from VEGF inhibitors which reduce vessel formation and increase vessel maturity but without affecting vessel size and lumen formation, which we observed with the dodecasaccharide.

## Discussion

For decades, studies of HS-ligand structure-activity relationships have been hindered by a lack of structurally-defined, site-specifically sulfated HS oligosaccharides. Here, using defined, differentially 6-*O*-sulfated dodecasaccharides, we show that the level of 6-*O*-sulfation, and indicatively the specific number of sulfates, along the uniformly 2-*O*- and *N*-sulfated HS chain plays an important role in determining the inhibitory properties of the oligosaccharide against FGF2-induced signaling and endothelial cell responses, *in vitro* and *in vivo*.

Some studies have suggested that HS binding to FGF2 does not depend on the HS sulfation pattern but requires correct spacing between adequately sulfated domains and/or that charge density is the critical determinant of biological behavior [15–16], while others have shown that there is a critical threshold number of 6S moieties that converts HS fragments from non-activating species to FGF2 signal-supporting sequences [11–14]. The findings of this study suggest the latter model and imply that a critical level of 6-*O*-sulfation determines the impact of an oligosaccharide on FGF2 biology. To address this question further, a novel synthesis strategy will have to be employed to generate a series of dodecasaccharides with a single 6S positioned in different disaccharides along the 2-*O*- and *N*-sulfated backbone.

We have shown that the presence or absence of a single non-reducing end 6S interconverts the inhibitory properties of [NSIS6S]-[NSIS]_5_ and [NSIS]_6_ as an inhibitor of CXCL8 or CXCL12, respectively [17]. The current study, taken alongside previously published IL-8 and SDF-1α data [17], further suggests that structurally-specific small changes in 6S level can regulate biological effects across a range of cytokine-mediated processes. Here the impact of the single 6S was less profound than the near on-off effect on IL-8 and SDF-1α [17] into a statistically significant change in potency. Our data suggest that certain chemokines manifest a high requirement for specific structural features in HS, whereas other cytokines, such as FGF2, are impacted significantly, but not absolutely, by this small structural change. Thus our data enables drug developers to focus on structurally critical moieties.

In this study we have described the anti-angiogenic and anti-tumor activity of [NSIS6S]-[NSIS]_5_ in different cancer models. The microvessel density in FGF2-overexpressing endometrial cancer xenografts was reduced to control levels by [NSIS6S]-[NSIS]_5_ with inhibition of FGF2 signaling in tumor blood vessels. Tumor growth was unaffected, in contrast to the effect of sunitinib suggesting that, despite overexpression of FGF2, FGF2-B9 tumors still significantly depend on VEGF-driven angiogenesis.

We show that [NSIS6S]-[NSIS]_5_ sensitizes ovarian cancer xenografts to cisplatin to the same extent as that seen with bevacizumab and sunitinib. However, the mechanism through which such sensitization occurs is unknown. These results open a new field for investigation of the mechanisms of synergy between natural or synthetic HS-based compounds and cytotoxic agents used in cancer treatment. In agreement with our data, a recently published study showed that low molecular weight heparin (LMWH; tinzaparin) at therapeutic doses changed the transcriptional profile in ovarian cancer cells and reversed resistance to cisplatin [24]. Since resistance to cytotoxic agents inevitably develops in ovarian cancer, chemotherapy-sensitizing low toxicity HS-based compounds would be of great benefit.

[NSIS6S]-[NSIS]_5_, when dosed in combination with ciplatin, reduced lumen formation and the size of vessels, distinguishing the biological effect from that seen with cisplatin-bevacizumab or sunitinib combination regimens. [NSIS6S]-[NSIS]_5_ inhibits multiple targets, e.g. FGF2 as we have shown in this study and SDF-1α [17]. In addition, VEGF_165_-induced endothelial cell behaviors were also affected by [NSIS6S]-[NSIS]_5_, although to much lesser extent. That FGF2 increases the size of blood vessels [25], SDF-1α induces large lumen containing vascular structures [26] and inhibition of VEGF leads to a more mature vascular phenotype [27], suggests that [NSIS6S]-[NSIS]_5_ may target FGF2, SDF-1α and VEGF_165_ in ES2 tumors leading to reduction of vessel size, density and lumen formation, while increasing vascular maturity. Of interest, our unpublished data have shown that SDF-1α is abundantly expressed in ES2 xenograft tumors.

In summary, our study has illustrated the potential that total chemical synthesis of HS oligsoaccharides has brought to the field. Defined positioning of 6S moieties and the ensuing biological studies has challenged the concept of charge density as the sole determinant of biological activity and further supports the proposition that structure-specific modifications offer strong prospects for the development of new oligoaccharide therapeutics to target mediators of resistance to licensed VEGF inhibitors.
